# Decoding the Tumor Microenvironment: Insights and New Targets from Single-Cell Sequencing and Spatial Transcriptomics

**DOI:** 10.3390/cimb47090730

**Published:** 2025-09-09

**Authors:** Shriya Pattabiram, Prakash Gangadaran, Sanjana Dhayalan, Gargii Chatterjee, Danyal Reyaz, Kruthika Prakash, Raksa Arun, Ramya Lakshmi Rajendran, Byeong-Cheol Ahn, Kandasamy Nagarajan Aruljothi

**Affiliations:** 1 Department of Genetic Engineering, College of Engineering and Technology, SRM Institute of Science and Technology, Kattankulathur 603 203, Tamilnadu, India; sp9705@srmist.edu.in (S.P.); sd2312@srmist.edu.in (S.D.); gc7124@srmist.edu.in (G.C.); dr6483@srmist.edu.in (D.R.); kp8418@srmist.edu.in (K.P.); raksaarun06@gmail.com (R.A.); 2 Department of Nuclear Medicine, School of Medicine, Kyungpook National University, Daegu 41944, Republic of Korea; prakashg@knu.ac.kr (P.G.); ramyag@knu.ac.kr (R.L.R.); 3 Cardiovascular Research Institute, Kyungpook National University, Daegu 41944, Republic of Korea; 4 BK21 FOUR KNU Convergence Educational Program of Biomedical Sciences for Creative Future Talents, School of Medicine, Kyungpook National University, Daegu 41944, Republic of Korea; 5 Department of Nuclear Medicine, Kyungpook National University Hospital, Daegu 41944, Republic of Korea

**Keywords:** TME, tumor micro-environment, signaling molecules, ECM, extracellular matrix, SCS, single-cell sequencing, ST, spatial transcriptomics, new targets

## Abstract

The field of oncology has been extensively studied to design more effective and efficient treatments. This review explores the advanced techniques that are transforming our comprehension of cancer and its constituents. Specifically, it highlights the signaling pathways that drive tumor progression, angiogenesis, and resistance to therapy, as well as the modern approaches used to identify and characterize these pathways within the tumor microenvironment (TME). Key pathways discussed in this review include vascular endothelial growth factor (VEGF), programmed cell death protein 1/programmed cell death ligand 1 (PD-1/PD-L1), cytotoxic T-lymphocyte-associated protein 4 (CTLA-4), and various extracellular matrix (ECM) pathways. Conventional methods of diagnosis have yielded sufficient knowledge but have failed to reveal the heterogeneity that exists within the TME, resulting in gaps in our understanding of the cellular interaction and spatial dynamics. Single-cell sequencing (SCS) and spatial transcriptomics (ST) are effective tools that can enable the dissection of the TME with the resolution capacity of a single cell. SCS allows the capture of the unique genetic and transcriptomic profiles of individual cells along with rare cell types and new therapeutic targets. ST complements this by providing a spatial map of gene expression, showing the gene expression profiles within the tumor tissue at specific sites with good accuracy. By mapping gene expression patterns at a single cell level and correlating them with the spatial locations, researchers can uncover the intricate networks and microenvironmental influences that contribute to tumor heterogeneity.

## 1. Introduction

Cancer remains one of the most formidable health challenges worldwide, complicated by factors arising from the intricate and evolving character of the tumor microenvironment (TME). TME exhibits a heterogeneous structure consisting of stromal cells, cancer cells, the extracellular matrix (ECM), immune cells, and various signaling molecules. Each component of the TME plays a role in promoting cancer progression and metastasis to various distinct organs [1,2]. The need to delineate the TME’s vast potential pertains to innovation in therapeutic strategy development and favorable clinical outcomes. A diverse range of cells and extracellular components makes up the TME. The ECM of the TME is reshaped by cancer-associated fibroblasts (CAFs), creating a supporting stroma that permits cancer cells to infiltrate and propagate across the surrounding tissues. The TME also contains immune cells that affect the behavior and growth of the tumor, such as regulatory tumor-associated macrophages (TAMs) and regulatory T-cells (Tregs) [3]. The endothelial cells and the vascular structures they generate not only provide nutrients and oxygen to the tumor but also generate routes for metastatic spread through angiogenesis. Some extracellular vesicles (EVs) and secreting factors (SFs), like cytokines, growth factors, and others, also affect the TME by sending signals between cancer cells and stromal parts. This causes tumors to grow and makes treatments less effective [4,5]. Considering this, it is apparent that modulating various aspects of the TME bears substantial therapeutic opportunity due to the system’s complicated nature. Indeed, strategies aimed at reprogramming immune cells, normalizing the ECM, or inhibiting angiogenesis are currently under active investigation [6,7]. For example, immune checkpoint inhibitors give an indication of the reactivation of the immune system’s dormant antitumor defenses, and ECM-targeting therapies are meant to break down the protective stroma around the tumor cells [8].

Recent advancements in cancer research underscore the critical nature of the TME in the progression of cancer and the resistance to therapy against it. As an example, CAFs have been found in up to 80% of stromal tissues in different cancer types [9,10]. They heavily influence the reorganization of the ECM and help tumors invade and spread [11]. Studies dealing with TAMs have shown that an excessive presence of TAMs is correlated with a poor prognosis in over 20 cancer types [12]. These statistics highlight the complexity and critical role of the TME, emphasizing the potential impact of targeted therapeutic strategies aimed at modulating the TME for improved clinical outcomes. However, conventional approaches often fail to capture the full heterogeneity and spatial organization of the TME. To overcome these limitations, advanced techniques such as SCS and ST provide powerful tools to characterize individual cell populations, uncover rare subtypes, and map their spatial distribution within tumor tissues. Thus, these technologies enable a more comprehensive understanding of TME biology and its impact on therapeutic outcomes.

### Diagnosis by Single-Cell Sequencing (SCS) and Spatial Transcriptomics (ST)

Cancer diagnosis by histopathology and immunohistochemistry (IHC) offers great information regarding the existence and spread of cancer, but fails to provide comprehensive details about the TME and its individuality. Most of these methods involve the general dissection of tissue samples, which hides important information regarding the relationships and spatial distribution of various cell subpopulations within the tumor and its surrounding stroma. Thus, to better understand the TME’s specifics, there is a need to fine-tune these methodologies [13]. Next-generation sequencing (NGS) technologies have revolutionized cancer diagnostics by enabling comprehensive genomic and transcriptomic profiling. However, even these advanced techniques often overlook the spatial context and single-cell heterogeneity that are pivotal in understanding the TME’s role in cancer progression. To address these limitations, SCS and ST have emerged as powerful tools. It is now possible to study the TME at a much higher resolution thanks to SCS. This lets scientists find different groups of cells and figure out what they do in relation to the tumor. At the level of a single cell, SCS can reveal genetically and transcriptomically defined subpopulations within the tumor. It can also find cell subtypes and signaling pathways that have not been described before, but can help cancer grow and become resistant to treatment. Detailed information is important for finding new therapeutic targets as well as comprehending tumor heterogeneity dynamics [14].

ST is another technique where spatial information is combined with gene expression data, offering a more complete understanding of the cellular structure of the TME. This technique maintains the original position of the cells and helps to determine how cell interactions and the local environment affect the activity of tumors. For example, ST can show where immune cells are positioned concerning cancer cells and how immune evasion works, and can then find targets for immunotherapy [15]. In the case of the TME, SCS and ST are complementary in their capacity to address the multi-layered nature of the concept. In contrast to SCS, which gives a detailed list of the sample’s cells and their molecular state, ST places these discoveries within the context of a specific cancerous mass. These technologies work together to make studying the TME more comprehensive. This helps provide a better understanding of the different signaling pathways that support tumor growth. This will help us come up with new treatments that can change the microenvironment, so that it rejects tumors instead of helping them grow.

ST has opened new doors in developmental biology by allowing researchers to study not just what genes are expressed, but also where they are expressed in tissues. Unlike single-cell RNA-seq, which loses spatial context, ST helps researchers to see how cells interact, organize, and change over time. This makes it powerful for studying processes like tissue patterning, organ development, and even TME. However, the technology is expensive, technically demanding, and currently lacks the sensitivity and resolution of single-cell approaches [16]. SCS is a powerful tool because it uncovers both inter- and intra-tissue heterogeneity, giving insights that bulk methods often miss. It also requires only small tissue samples, which can be collected from different sites within an organ. However, the method has clear limitations—working with tiny amounts of material makes it highly sensitive to degradation, contamination, and sample loss. The necessary amplification can introduce biases and errors, and if barcodes are misread, valuable data may be lost [17].

## 2. Therapeutic Targets in the TME

### 2.1. Components of the TME

The TME hosts different kinds of cells, signaling molecules, vesicles, and ECM, among other types of cellular substances, which are discussed in detail below.

The various kinds of T-cells, like killer T cells and cytotoxic T cells that are present in the TME, affect the initiation, progression, and metastasis of tumors in the following ways. Tregs are widely distributed in the TME and promote the development and metastasis of malignancies by inhibiting the immune responses that combat the tumor. Certain antibodies, such as OX-40, GITR, and CD-40, can be utilized to boost T-cell responses that are specific to a certain antigen [18]. The CD25 antibody can decrease the quantity of Treg cells or impede Treg activity. Cytotoxic T lymphocytes, namely those expressing the CD8+ marker, identify and recognize atypical tumor antigens that are present on cancerous cells and identify them as targets for annihilation [19].

B cells help in the formation of tertiary lymphoid structures and tumor infiltration. These structures act as prognostic markers in some cancers. B cells are also known for storing memory of previous infections; these are memory cells that use the knowledge of the previous infection to fight the tumor. B cells produce cytokines like IL-10 and transforming growth factor β (TGF-β) to aid in tumor progression, while also acting as tumor suppressors due to the production of IFN-γ. B cells also inhibit events such as TGF-β-mediated conversion of FoXP3+ cells that can lead to metastasis [20].

Natural killer cells (NK Cells) make up around 15% of all lymphocytes in circulation. These cells can destroy tumor cells of various kinds in the bloodstream, but are not as successful in eradicating tumors in the TME. Studies have shown that NK cells may differentiate between normal and modified self by using antagonistic receptors unique to major histocompatibility complex (MHC) class I molecules and activating receptors that detect ligands that indicate cellular stress [19].

TAMs are vital constituents of the innate immune system that regulate immune responses by engulfing pathogens. They can be seen in regions where the basal membrane is disrupted and breached in the initial stages of some breast cancers. It suggests that TAMs may be involved in the infiltration of cancer cells into the adjacent healthy tissue.

Macrophages are categorized into two classes, namely M1 macrophages, which are inflammatory, and M2 macrophages, which can suppress the immune responses and contribute to the healing of wounds. The TME encourages the M2 phenotype by creating low oxygen levels (hypoxia) and releasing cytokines (such as IL-4) to facilitate tumor development and advancement [21].

Dendritic cells (DCs) are a unique type of immune cell that display both pro-tumorigenic and antitumorigenic actions. Their role is to distinguish, secure, and ferry antigens to T cells in secondary lymphoid organs [22].

Neutrophils constitute approximately 70% of the leukocytes present in the bloodstream. TGF-β can influence the neutrophils to acquire an N2 phenotype, which promotes tumor progression. Thus, inhibiting TGF-β function can cause neutrophils to acquire an N1 phenotype, exhibiting antitumor-like properties in both laboratory settings and living organisms [19].

Adipocytes determine the nature of the TME by releasing hormones, metabolites, growth factors, enzymes, and cytokines. Current in vitro, in vivo, and clinical studies demonstrate that adipocyte-derived factors alter their characteristics as cancers progress and that tumor cells can also significantly affect neighboring adipocytes. Peritumoral adipocytes, known as “cancer-associated adipocytes” (CAAs), are characterized by a decrease in mature adipocyte development markers and an increase in the metabolic reprogramming of tumor cells [23].

A range of pro-inflammatory cytokines and chemokines is produced by CAFs, which function as feedback regulators of cancer cell activity. CAFs can constitute up to 80% of the fibroblasts in a tumor [24]. The development factors and cytokines TGF, HGF, SDF-1, and IL-1, which are continuously secreted by CAFs, boost the growth and spread of surrounding tumor cells and promote angiogenesis in a variety of malignancies, including breast, pancreatic, ovarian, and liver cancers, and trigger an inflammatory response that promotes tumor development. The fibroblasts closest to the tumor are the most active [25].

Although the cells occupy about 65–70% of the TME, there are other valuable components, like the ECM, which comprises laminin, proteoglycans, collagen, fibronectin, elastin, and hyaluronic acid. Higher levels of collagen expression are correlated with an increased risk of metastasis and stiffer tumors [26,27]. Laminins form an important component of the basement membrane. During tumor progression, the basal membrane can lose its adherence and rupture. Quite different from collagen and laminin are proteoglycans, whose breakdown seems to hold prognostic implications; for example, higher levels of aggrecan have been found in prostate cancer tissue than in normal tissue [27,28]. In several types of cancer, the ECM exhibits elevated amounts of elastin, which can potentially have both beneficial and detrimental influences on the proliferation of tumors. For example, neutrophil elastase plays a crucial role in promoting invasion and metastasis. Elastin-like peptides and elastin-derived peptides can be used to suppress tumor progression [28,29].

Exosomes can stimulate the progression of tumors in glial cells by inducing autophagy, which encourages the M2 phenotype of macrophages. In these cells, the release of exosomes results from low-oxygen or hypoxic conditions in the TME. Exosomes have also been proven to hinder cell death due to the significant presence of miRNA-210-3p, which also enables the interphase transition from growth phase (G1) to synthesis phase (S). Due to the presence of activated TGF-β1, exosomes derived from prostate cancer tissue can initiate the transformation of fibroblasts into myofibroblasts. Similarly, exosomes retrieved from the breast cancer cells are able to transform mesenchymal stem cells into myofibroblasts. Tumor exosomes can enhance the enlistment of fibroblasts, hence boosting tumor angiogenesis. Additionally, cancer cells transfer EGFR—which is encapsulated within a membrane—to endothelial cells by exosomes [30,31]. The EVs’ cargo, such as EGFR, can suppress the immune system in macrophages, while TGF-β, programmed cell death ligand 1 (PD-L1), and tumor-associated antigens can cause cell death in CD8+ cells and induce the activation of these cells, respectively. Other EV cargos, such as PKM2, promote tumor development by causing the monocytes to undergo division into macrophages, showcasing the M2 phenotype [32]

### 2.2. Pathways Involved with the TME

The pathways of the TME are typically classified based on the cell or ECM where the pathway takes place. Predominantly, the proteins produced by the cell, like vascular endothelial growth factor (VEGF) and cytotoxic T-lymphocyte-associated protein 4 (CTLA-4), undergo intracellular pathways, whereas there are pathways like SRC and FAK that take place outside the cell in the matrix.

#### 2.2.1. VEGF

The VEGF, a protein with a molecular weight of 45-kDa, is mainly produced by the VEGFA gene found on chromosome 6. The signaling system plays a key role in regulating angiogenesis, the formation of new blood vessels, which is crucial for tumor growth and spread [33,34,35]. The VEGF family includes placental growth factor (PlGF), VEGF-A, VEGF-B, VEGF-C, and VEGF-D. Among these, VEGF-A has been the subject of extensive scientific research. It stimulates the growth of blood vessels that supply the tumors with essential nutrients and oxygen, fostering their expansion [36,37,38]. This heightened angiogenesis facilitates the invasion of cancer cells into surrounding tissues and provides a potential avenue for metastasis via vascular permeability, enabling cancer cells to escape into nearby tissues, contributing to the aggressiveness of the disease [30,31]. VEGF can also be produced in the ECM by matrix metalloproteinase-9 (MMP-9) to activate an angiogenic switch that supports tumor proliferation. This creates a loop where the secreted VEGF binds to the VEGF-receptor (VEGFR) on the surface of endothelial cells while increasing the expression of proteases like MMPs. MMPs help create niches for the new vessels to thrive by proteolyzing the ECM around the vessels and by producing pro-angiogenic compounds [39,40]. Consequently, tumor angiogenesis is enhanced, resulting in the development, proliferation, and migration of endothelial cells. VEGF induces epithelial–mesenchymal transition (EMT) by means of an autocrine loop [41], showing its involvement in both tumor angiogenesis and the initial spread of malignant cells beyond the epithelial layer. Cytokines and growth factors, such as epidermal growth factor receptor/human epidermal growth factor receptor 2/erythroblastic leukemia viral oncogene homolog 1,2 (EGFR/HER-2/ErbB1,2), and insulin-like growth factor I receptor (IGF-IR), have been found to regulate the expression of VEGF and thereby, angiogenesis as well [42].

#### 2.2.2. Hypoxia-Inducible Factor 1-Alpha (HIF-1α)

HIF-1α, a protein encoded by the gene HIF-1α, is found on the longer arm of chromosome 14 in humans. The HIF-1α gene codes for the transcription factor HIF-1, which is often induced by reduced oxygen levels or, to a lesser extent, by cytokines, growth factors, or circulatory factors [43].

The TME is usually hypoxic in nature due to the rapid growth of tumors, which is not matched by the growth of vascular systems around the tumor. To combat this hurdle, the cancer cells opt for metabolic rewiring to quench their oxygen-intensive activity that makes way for cell proliferation, metastasis, and invasion [44,45]. The body inadvertently ends up promoting tumor growth by inhibiting antitumoral activities as a way of protecting its tissues from chronic inflammation [46,47].

**Figure 1:**
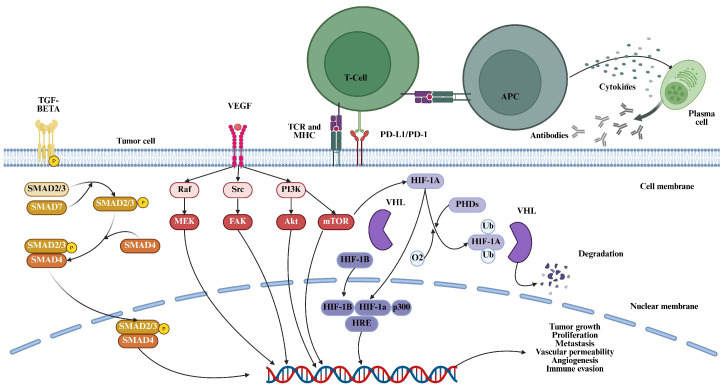
A compilation of all the pathways involved in the TME. In this image, receptors of various pathways like VEGF, HIF-1α, TGF-β, PD-1/PD-L1, CTLA, and NF-κB pathways are implicated in the proliferation, metastasis, and growth of cancer cells. APC, hypoxia response element (HRE), MHC, PHD proteins, pVHL, T cell receptor (TCR).

HIF-1α overexpression is linked to tumor growth and apoptosis prevention by overcoming hypoxia. HIF-1α, with the help of VEGF, overcomes hypoxia by the formation of new blood vessels to combat the lack of oxygen caused by a scarcity of blood vessels that carry the necessary nutrients to the TME. Phagocytosis by macrophages is blocked by transcription of the CD47 gene that is activated by the HIF-1 during hypoxia [48,49]. Under normoxic conditions, the α subunit is degraded by oxygen-dependent proteolysis and ubiquitylation due to the recognition of the von Hippel–Lindau tumor suppressor protein (pVHL), as shown in Figure 1. For interacting with pVHL, the HIF-alpha subunits need to be hydrolyzed by an iron-dependent enzyme, HIF-PH, which belongs to a class of prolyl hydroxylase domain proteins (PHDs) [50,51]. Mutations in the VHL lead to disrupted HIF-1α ubiquitylation, which gives way to increased promotion of improperly regulated angiogenesis [52]. PHD activity is inhibited as a hypoxic response, giving way to modified expressions of the VEGF, erythropoietin, hypoxia-inducible lipid droplet-associated (HILPDA) protein, and other hypoxia-response genes, which help the cell adjust to hypoxia [53].

#### 2.2.3. TGF-β

The TGF-β signaling system influences cell proliferation, differentiation, adhesion, senescence, and death of cancer cells [54,55,56]. TGF1, TGF2, and TGF3 are homodimers that are secreted as members of the TGF protein family. The most prevalent isoform is TGF-β1, a 44-kDa protein expressed by the TGF-β1 gene on chromosome 19. The TGF pathway plays two distinct functions in the TME—it promotes tumor growth in late-stage malignancies and has potential cancer suppressor activities during carcinogenesis. In later stages of cancer, TGF-β regulates EMT and metastasis [57]. Certain evidence can validate the tumor suppressor action of TGF-β, such as the production of angiopoietin 4 in response to TGF-β by receptor-negative breast carcinoma cells in early tumors, which increases the dissemination of these cells as they migrate into the lung capillaries and enter the circulation [58]. Additionally, TGF-β signaling drives a non-inflammatory program in intestinal epithelial macrophages, which show a marked reduction in inflammatory response to stimuli but retain bactericidal activity [57].

The TGF-β signaling pathway is necessary to create a TME that suppresses the immune system while supporting the traditional hallmarks of cancer, like growth, invasion, metastasis, resistance, and recurrence. This suppressive nature inhibits the growth of cytotoxic components like T cells, NK cells, and B cells, while simultaneously promoting Tregs and myeloid-derived suppressor cells (MDSCs). Furthermore, it also enhances programmed cell death protein 1 (PD-1) production on CD8+ T cells, contributing to the depletion and subsequent impairment of the immune response in [59,60].

TGF-β type I and II receptors are the components of the tetrameric receptor complex to which activated TGF-β ligands bind. TGF-βRII activates TGF-βRI, leading to SMAD2/3 phosphorylation. These then join with SMAD4 and move into the nucleus, where they bind specific DNA regions to regulate gene expression [61].

Apart from the conventional signal transduction of TGF-β, the noncanonical signaling pathways have significant implications in the development of illnesses. TGF-β ligands have the ability to stimulate non-SMAD signaling pathways, such as mitogen-activated protein kinase (MAPK), Hippo, AMP-activated protein kinase (AMPK), and phosphoinositide 3-kinase (PI3K)/AKT signaling, through non-canonical routes [62].

#### 2.2.4. PD-1/PD-L1

The PD-1/PD-L1 signaling pathway regulates immune responses, which cancer cells exploit in order to evade detection. PD-L1 is a 33-kDa glycoprotein with 290 amino acids and is encoded by CD274, whereas PD-1 is a 55-kDa receptor with 288 amino acids and is encoded by the PDCD1 gene. To assist in regulating T cell activity during immune responses, PD-L1 and PD-L2, which are present on immune cells such as T cells, B cells, macrophages, and DCs, bind to PD-1 [63]. The binding of PD-1 to PD-L1/PD-L2 initiates inhibitory signals within the T cell that result in the suppression of T cell activity. This suppression includes reduced proliferation and a diminished immune response. Prolonged exposure to PD-1/PD-L1 signaling may lead to T cell exhaustion, a state where T cells lose their normal function. This exhaustion is characterized by a decrease in their overall functionality. Tumor cells exploit the PD-1/PD-L1 pathway to their advantage, using it as a mechanism to evade cytotoxic T cell-mediated killing. By leveraging these inhibitory signals, tumors can escape the immune system’s attack, allowing them to persist and grow [64].

The expression of PD-L1 is monitored through two primary mechanisms—the upregulation of PI3K-Akt kinases or the secretion of IFN-γ, which initiates the pathway [65]. After the initiation, two types of immune resistance pathways take place—innate immune resistance type I and innate immune resistance type II. Under type I immune resistance, downregulation of PTEN leads to activation of PI3K-Akt signaling, driving PD-L1 expression in the glioblastomas. Under type II, certain lymphomas and lung cancers drive PD-L1 expression through upregulation of STAT3 and ALK signaling pathways. STAT3 activation is modulated by pro-inflammatory cytokines such as IL-6, contributing to tumorigenic macrophage polarization and immune suppression [66]. In certain tumors, PD-L1 expression is triggered by pro-inflammatory IFN-γ released from tumor and stromal cells. This IFN-γ suppresses the antitumor activity of CD8^+^ cytotoxic T cells, resulting in adaptive immune resistance [67].

#### 2.2.5. CTLA-4

CTLA-4 belongs to the CD28–B7 immunoglobulin superfamily [68]. In humans, CTLA-4 is found on band q33-q34 of chromosome 2. Exons 1 through 4 of the CTLA-4 gene encode the three primary components of the CTLA-4 protein—a transmembrane region, a ligand-binding domain, and a cytoplasmic tail. Higher CTLA-4 mRNA levels in breast cancer patients are associated with more advanced disease and metastasis to axillary lymph nodes, according to studies [69,70].

The interaction between B7-1 (CD80) and B7-2 (CD86) molecules on the antigen-presenting cells (APCs) and CD28 molecules on T cells triggers signaling cascades within the T cells. Sufficient levels of CD28:B7-1/2-binding result in the proliferation of T cells, enhanced T-cell survival, and the upregulation of genes associated with cell survival. CTLA-4 competes with the costimulatory receptor CD28 for binding to the B7 ligands (CD80/CD86) on APCs. CTLA-4 has a much higher affinity for B7 than CD28, so it can outcompete CD28 and prevent the costimulatory signal required for full T-cell activation [71]. In addition to competitive inhibition, CTLA-4 can also deliver direct negative signals to T cells through its cytoplasmic domain. This inhibits IL-2 production and cell cycle progression, thereby suppressing T-cell responses. High expression of CTLA-4 on Tregs can downregulate B7 expression on APC, inhibiting the activation of effector T cells [72].

The following are the pathways of the ECM.

#### 2.2.6. SRC and FAK

The activation of Src and FAK plays a crucial role in the control of multiple downstream signaling pathways, particularly those associated with angiogenesis. Src and FAK have the capacity to influence the metastasis, proliferation, and survival of endothelial cells, which are fundamental processes in the development of neovascularization [73].

The activation of VEGFRs can result in the activation of Src, which is a non-receptor tyrosine kinase. The Src protein is recruited to the complex formed by activated VEGFR and undergoes phosphorylation, leading to its activation [74].

The activation of Src leads to the phosphorylation and subsequent activation of FAK, a tyrosine kinase located in the cytoplasm. FAK, also known as focal adhesion kinase, is frequently linked to integrin-mediated signaling pathways and focal adhesions, playing a crucial role in cellular phenomena such as migration and survival [75,76].

#### 2.2.7. NF-κB Pathway

The nuclear factor kappa-light-chain-enhancer of activated B cells (NF-κB) pathway is one of the key regulators of the TME, modulating the production of pro-inflammatory cytokines, chemokines, and other mediators by various cell types like tumor cells, stromal cells, and immune cells [77]. This shapes the immunosuppressive and pro-tumorigenic TME. NF-κB signaling plays a crucial role in polarizing TAMs toward a pro-tumor M2 phenotype, which can suppress antitumor immunity and promote angiogenesis, metastasis, and therapy resistance [78]. The physical and molecular properties of the ECM can modulate NF-κB signaling in the TME. For example, a stiff ECM enhances NF-κB activity, leading to increased production of inflammatory mediators that further remodel the ECM. This creates a feedforward loop that supports tumor progression. A soft ECM enhances drug resistance by increasing NF-κB and decreasing pro-apoptotic JNK signaling. On the other hand, the ECM can also influence NF-κB signaling—ECM components like fibronectin and collagen can activate or suppress NF-κB, respectively. Conversely, NF-κB regulates the expression of ECM remodeling enzymes like MMPs, further modifying the transcription [79] (Figure 1).

### 2.3. Protein Targeting

Given below is the Table 1 depicting the list of all the proteins in the TME, along with their functions and respective pathways.

Tirosh et al. conducted the first large-scale single-cell RNA sequencing (scRNA-seq) analysis of the TME by profiling CD45^+^ and CD45^−^ cells from 19 melanoma patients. Their work revealed distinct T cell exhaustion programs unique to individual patients, providing insights potentially valuable for immunotherapy [91]. Similarly, Chung et al. analyzed tumor and immune cell populations—including T cells, B cells, and macrophages—in 11 breast cancer samples [92]. Recent advances in SCS and ST have significantly enhanced our understanding of TME heterogeneity, as seen in Table 2. These technologies allow precise identification of immune cell subsets (e.g., distinct Treg or TAM populations), spatial mapping of cytokine/chemokine gradients, and localization of pathway activations (e.g., VEGF, HIF-1α) within tumor niches. Such insights have facilitated the identification of novel, spatially restricted therapeutic targets within the TME that conventional bulk profiling could not resolve [93,94].

## 3. Diagnostics

For the past several decades, cancer diagnosis has predominantly relied on classical histopathological techniques, like microscopy of stained tissue sections, to visualize the morphological changes that are characteristic of malignant tumors. IHC has further refined diagnostic capabilities by using antibodies to detect specific antigens in the cells of a tissue section. IHC can assist in determining the biomarkers that could define the cancer type and produce the prognosis. For instance, the determination of hormone receptors (ER, PR) and HER2, which are often employed when diagnosing breast cancer, employs IHC [101].

Various imaging procedures, like ultrasound, MRI, CT, and PET scans, also form part of the diagnostic process for effectively diagnosing medical conditions. These imaging-enhancing technologies aid in visualizing tumors within the body and are useful in diagnostics, cancer staging, and evaluation. Although historical methods play an essential role in cancer diagnosis, they have drawbacks, including a lack of clear molecular information on tumor behavior. This space has been gradually filled by improvements in NGS technologies [102].

The aim of NGS is to sequence millions of DNA fragments at the same time and, therefore, identify a wide range of genetic changes. Various NGS techniques have been used recently for diagnosis, which involve whole genome sequencing (WGS), whole exome sequencing (WES), targeted sequencing, and RNA sequencing. WGS provides a comprehensive overview of the mutation profile within cancer cells, as it involves the sequencing of the complete genome. Point mutations, which are made up of single-nucleotide variants (SNVs), insertions, deletions, sequencing-induced variations, copy number variations, and structural rearrangements, are all detected by WGS [103]. WES targets the exons specifically, which are the working parts of the genome. Since most of the known disease-causing mutations are limited to these zones, WES saves money when compared to WGS, but at the same time, it is quite helpful for providing insight into the genetic aspects of cancer [104]. Targeted sequencing is an approach that involves stripping a pre-selected number of genes that are already believed to be linked to cancer in a sequential manner. Gene panels provide 90% sensitivity for the discovery of mutations in areas of focus and are regularly used in clinical analysis [105]. RNA-seq looks at the transcriptome and evaluates gene expression, fused genes, divergence, alternative splicing, and non-coding RNA. It assesses and characterizes the functional status of the tumor, which consequently aids in the identification of possible therapies [106].

NGS has filled significant gaps left by classical diagnostic methods by offering detailed molecular insights into cancer, which can guide personalized treatment strategies. However, the complexity and heterogeneity of tumors necessitate even more precise techniques, such as SCS and ST. SCS offers significant benefits for TME diagnostics and therapeutic approaches by allowing the identification of individual cellular states, uncovering rare cell subpopulations, and providing insights into the mechanisms of tumorigenesis, metastasis, and immune evasion. This detailed resolution is crucial for identifying drug-resistant clones, developing individualized treatment strategies, and detecting biomarkers [107]. Nevertheless, SCS is constrained by many significant issues, including an absence of spatial context, technical noise from amplification, and disruptions that affect sensitivity. This indicates that it cannot disclose the physical relationships among certain cells inside a tissue. Moreover, the sample throughput may fail to include the whole spectrum of tumor heterogeneity, and SCS procedures are often costly and intricate [108].

Conversely, ST is characterized by its ability to maintain the spatial arrangement of gene expression within tissue, enabling researchers to correlate molecular markers with specific anatomical or microenvironmental sites. This capability is essential for comprehending the interactions between tumor cells and their surroundings, delineating invasion patterns, and seeing immune infiltration [109]. The therapeutic significance of ST may be augmented by using it on both fresh and fixed samples, often in combination with protein or imaging analysis. The constraints include restricted gene coverage in imaging-based systems, elevated costs, and analytical difficulties in combining ST data with SCS or other omics technologies [110]. Moreover, many ST platforms characterize areas that include multiple cells, resulting in limited single-cell resolution. Notwithstanding these constraints, ST provides a geographical context that is essential for assessing the functional and therapeutic relevance of complex tumor ecosystems, which uniquely enhance SCS [107].

### 3.1. Single Cell Sequencing

SCS is one of the most sophisticated methods of NGS that enables the genomic, transcriptomic, and epigenomic profiling of single cells. In contrast to other mass sequencing methods that study a pool of cells, SCS provides specific data on the variation of genetic and phenotypic characteristics within a tumor through single-cell analysis [111].

**Figure 2:**
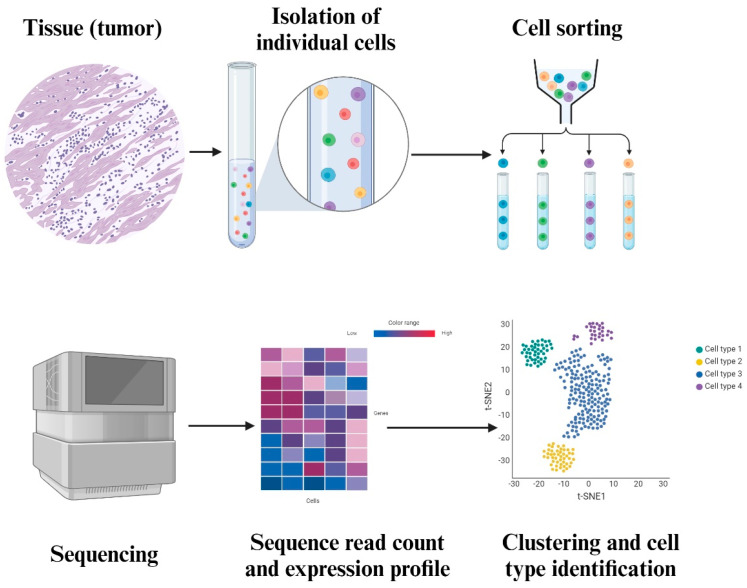
This figure depicts the process of SCS. The tumor tissue is fixed and is then isolated into individual cells in a suspension medium. Each individual cell is sequenced, and the read count is used to create an expression profile heatmap. This heatmap is converted into a cluster map to find out more about the cell types that are within the tissue.

Tumors are heterogeneous, consisting of genetically and phenotypically diverse cell populations. Unlike conventional high-throughput sequencing, SCS offers unprecedented resolution, identifying single-cell populations and single-nucleotide polymorphisms often missed by mass approaches [95]. This resolution is especially significant in the TME, where clinically relevant subclones that are rare may promote tumor development, treatment resistance, or recurrence. One of the major strengths of SCS is its ability to demonstrate the heterogeneity of tumors. As shown in Figure 2, SCS allows the identification of different subsets of cells within a tumor that are characterized by specific genetic changes and activation status [91].

SCS allows for lineage tracing, mapping the development and the evolutionary history of individual cancer cells. This provides crucial insights into tumor characteristics and therapeutic responses, shedding light on cancer cell origins and regulatory processes. Moreover, SCS captures dynamic processes such as cell differentiation and plasticity, which are essential for understanding cancer stem cell biology and its contribution to tumor maintenance and relapse [96]. Importantly, SCS enables the detection of diverse genomic alterations, including single-nucleotide variants (SNVs), insertions, deletions, copy number variations (CNVs), and structural variations. Collectively, these alterations are hallmarks of chromosomal instability (CIN), a major driver of tumor heterogeneity and cancer evolution [97]. By integrating these genomic changes within the broader context of CIN, SCS not only charts the mutational signature of cancers but also helps identify therapeutic vulnerabilities. In addition to genetic alterations, SCS can profile epigenetic modifications such as DNA methylation and chromatin accessibility, which regulate gene expression programs critical to tumor progression. Together, these layers of information offer a multidimensional view of cancer biology, deepening our understanding of both genetic and epigenetic contributors to malignancy [112].

Functional characterization of the TME is also performed in SCS, which mentions immune cells, stromal cells, and cancer stem cells. Describing these complex subpopulations and their relationships will help dissect the TME and design ways to boost antitumor immunity [92]. SCS integrates scientific tumor biology with clinical applications, providing diagnostic accuracy and therapy direction.

### 3.2. Spatial Transcriptomics

ST is an innovative technique for molecular profiling that enables scientists to measure the complete gene activity in a tissue sample and identify the exact regions where this activity is taking place. This technology is currently facilitating the exploration of novel information, thereby assisting scientists in attaining a more holistic comprehension of biological processes and disease. Unlike SCS, which compromises spatial context during cell separation, ST enhances molecular analysis by maintaining the tissue’s architecture.

**Figure 3:**
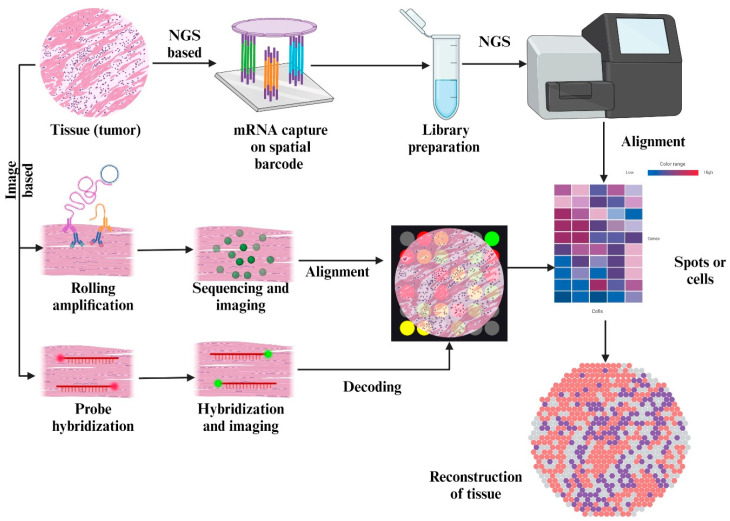
This figure depicts the process of ST. The tumor tissue is sequenced either through an image-based approach or an NGS-based approach. The result obtained is overlaid on a microarray of all expressed genes to produce a heat map detailing all the cells in the tissue. This tissue is rendered and reconstructed back, complete with sections detailing the distribution and nature of cells in the tissue.

Transcripts from tissues that have been preserved in formalin-fixed, paraffin-embedded, or sectioned frozen forms can be detected either by directly targeting specific regions of interest using photoactive in situ hybridization probes or by capturing them on slides using spatially localized probes. This allows for the spatial resolution of mRNA expression in tissue sections. Sequencing and analyzing cDNA libraries are employed for the quantification of expression levels. The expression of 1200–21,000 protein-coding genes may be statistically evaluated and correctly mapped using various methods. As shown in Figure 3, this allows for the extraction of transcriptional information from tissue slices in a single readout.

This novel technique is increasingly useful to obtain an understanding of a heterogeneous TME. The multifaceted nature of the TME, along with its cross-talk mechanisms, indirect extracellular signaling, and the various secretomes present in the ECM, like hormones, cytokines, and enzymes, causes drug resistance. ST aims to combat the loss of spatial information in SCS, where it fails to account for the incredible variation and heterogeneity present in tumors and the TME. This spatial resolution is a crucial advantage for clinical diagnostics, as ST may assist in classifying tumor margins, identifying invasive niches, and enhancing pathological annotation beyond the constraints of traditional histology. ST technology is the ideal tool for clinical applications in need of higher sensitivity and accuracy [98]. From a therapeutic perspective, ST can identify the geographical expression of resistance-related pathways, including VEGFA, TGF-β signaling, and PD-L1. This information may guide the creation of targeted therapies. In the field of developmental biology, ST is used to visualize stages of embryonic development and organogenesis in the context of dynamic changes to the gene expression profiles and cell types over the course of the different stages [99]. Spatiotemporal understanding of the TME involves the construction of a complete spatial map for the intratumoral transcriptomic profile, which details the expression of genes in comparison of normal cells to tumor cells. Such deep spatial profiling in combination with multi-modal analysis of the tissues is necessary to elucidate novel drug targets and produce a relevant, accurate reductionist prediction model [100] (Figure 2 and Figure 3).

## 4. Future Directions

SCS and ST are altering the future direction of cancer research and treatment, thanks in part to other omics technologies, including proteomics, metabolomics, and epigenomics. These multidimensional methods provide us with a full picture of the biology of tumors and their surroundings, which helps us find targets for treatment and learn more about how diseases progress. NGS tools have made a big difference in precision oncology by finding mutations that can be treated and helping doctors decide how to treat cancer [113,114,115]. It has been observed routinely that patients who receive personalized, precision therapy have better clinical outcomes [116].

Several study papers have talked about how bioinformatics and machine learning algorithms are improving [117,118], which is essential for looking at, putting together, and making sense of multi-omics data to find new biomarkers, therapeutic targets, and ways that cancer cells can fight treatment (Table 3). Researchers can easily navigate the huge amounts of data produced by high-throughput technologies with these advanced computing tools. This makes it easier to apply study findings in clinical settings. Using these tools makes it much more likely that SCS and ST technologies will be used in regular clinical practice. This will allow for more personalized and effective cancer treatments based on a better understanding of how the disease works at the molecular and biological levels.

Setting up standard procedures for collecting samples, processing them, testing them, and analyzing the data is very important for bridging the gap between study and clinical use [119,120]. These rules ensure that technologies like NGS can be used consistently and accurately in the diagnosis and treatment of cancer. They let doctors use genetic information about each patient to make better treatment choices. Researchers and doctors can use genomic data to help patients receive better care and advance precision medicine in oncology by following data management standards and guidelines like those promoted by the Innovative Medicines Initiative (IMI) and the Clinical Genome Resource (ClinGen). Standardizing how samples are collected and analyzed not only makes research easier, but it also makes it easier to combine genetic data with clinical data. This will help the development of personalized cancer treatments in everyday clinical practice [121].

SCS and ST make personalized medicine possible. This changes the way cancer is treated by giving doctors exact information about the TME so they can tailor treatments to each patient’s needs while reducing side effects [122,122,123,124]. These tools help us learn more about how tumors are different, how the TME interacts with them, how to find biomarkers, how to find therapeutic targets, and how to stop drugs from working [95,111]. By giving a clear picture of the immune environment around tumors, SCS and ST help find immunotherapeutic targets and immune resistance mechanisms, which are very important for making immunotherapies work better. The type of treatment for disease progression is determined by learning about the return rates as part of looking at the types of cells in tumors [122,123]. Also, progress in finding biomarkers through SCS and ST makes it possible to find new biomarkers for early detection, predicting prognosis, and estimating how a treatment will work. In the end, these tools help fight drug resistance, which makes cancer treatments more effective.

Scientists are looking into new targets in the TME, focused on molecules like LAG-3, TIM-3, and TIGIT instead of traditional immune checkpoints like PD-1/PD-L1 and CTLA-4 [125]. Researchers are also looking into ways to change TAMs from being pro-tumorigenic to antitumorigenic, with the goal of making treatments work better [126]. At the same time, studies into CAFs are looking into how the ECM affects the development of cancer to create more effective treatments [127]. Finding hypoxia-related factors in the TME, like carbonic anhydrase IX (CAIX), is also being investigated to improve treatment results by changing the acidic microenvironment and making it easier for immune cells to kill cancer cells [126].

Moreover, using SC and ST, we are able to predict the outcome and trajectory of cancer, along with the pathways that are to be affected by the development of the TME. In a study conducted by Zhu et al., the usage of single-cell RNA sequencing (scRNA-seq) and ST helps track the path of invasiveness of lung adenocarcinoma (LUAD) from an in situ carcinoma to an invasive one using a variety of markers, including gene expression patterns of different cell types present in the TME samples and gene sets of pathways that the most prolific cells are highly implicated in. scRNA-seq revealed that invasive development of LUAD is correlated with transcriptional changes that are specific to different cell types based on differentially expressed genes. The initiation of the invasive process and metastasis was attributed to a specific cell type, and a commonly enriched pathway was found through which cancer progression can be tracked. Investigation of distinct TME subpopulations during the invasive process of LUAD revealed that the involvement of the TGF-β pathway is crucial in invasion and metastasis, with the TGF-β signaling pathway-related genes being highly present in the central cancerous region. Tissue sections of cells in the various stages of the process were used for ST to reveal the spatial distribution of Tregs in cancer regions, and the abnormal presence of cancer blood vessels in the region contributed to the transition of the process. It was noted that the tracking of invasive LUAD in tandem with the TME using ST and scRNA-seq might be game-changing for targeted therapy development. Personalized targeted therapy remains a possibility through the analysis of tumor heterogeneity and spatial information from a patient’s ultra-specific tissue samples [128].

Prediction of TME status and the clinical response of patients to immunotherapy using models trained on tissue samples of patients opens new doors for more noninvasive methods of tracking and monitoring responses to chemotherapy. Current approaches to risk stratification and response to treatment predictions fail as prognostic models, as they fail to provide reliable and accurate predictive information across large swathes of the patient population due to a lack of sensitivity to variation within the population, even across similar stages of cancer. This may be attributed to an inadequate availability of tumor tissue rich in intratumoral heterogeneity. Deep learning and machine learning models trained on radiological images that correlate diagnosis, staging, and treatment response represent the next step forward in establishing a noninvasive method for prognostic prediction and clinical diagnosis [129].

Three-dimensional (3D) organoid cultures provide a promising alternative for the prediction and evaluation of cancer patients’ response to immunotherapy, based on the TME, which is not widely explored in conventional 2D cultures and imaging systems. Assessment of the TME as a whole is currently unstructured and inefficient due to a lack of useful models. Understanding the connection between the transcriptomic nature of cancer cells and the TME using scRNA-seq is significant for the early diagnosis and development of personalized treatment strategies that are both predictive and accurate [130]. One study by Zhang et al. employed the use of this method in order to derive an overview of the TME present in esophageal squamous-cell carcinoma (ESCC). This helped provide a few common expression programs that differed in terms of gene expression and pathway involvement. Investigation of the results helps identify several markers that are linked to a better survival rate, along with conditions that might warrant precision or individualized therapy for ESCC patients. It was found that mucosal program expression levels in patients were correlated with a better prognosis, with an increased immunotherapy response due to the recruitment of DC into the TME, which increased antitumoral immunity. This result also yielded markers for the program that may be utilized to diagnose and treat patients faster [131].

**Table 3:** Data regarding the previous studies conducted by scientists targeting various proteins of the TME.

Study and Scientists	Targeted on	Results
Review of the TME in basal and squamous cell carcinoma; Elizabeth Chiang et al. [132].	TME in basal and squamous cell carcinoma.	TME interactions promote tumor growth and progression. Immunotherapeutic agents like vismodegib and cemiplimab treat BCC and SCC.
Integrated analysis of TME using reconfigurable microfluidic platform; Nan Sethakorn et al. [133].	Elucidating the pathways underlying the roles of the TME and developing new TME-directed therapies for cancer treatment.	Stacks platform supports multicellular culture and integrated cellular analysis. Enhances TME research; potential for clinical translation and novel insights.
Immune modulation in TME; Zimu Deng et al. [134].	Immune escape mechanisms in the TME to improve cancer immunotherapy. Targeted approaches include CAR T-cell therapy, immune checkpoint blockers, TCR-based cell therapy, cancer vaccines, and oncolytic viruses.	Combines information from 16 original research studies and 2 reviews to highlight the role of the immunosuppressive TME in the initiation, spread, and resistance to treatment of cancer.
Targeting the TME for improving therapeutic effectiveness; I-Tsu Chyuan et al. [135].	Exploring molecular and cellular factors in the TME to uncover resistance mechanisms and develop new combination strategies for cancer immunotherapy.	Reviews advancements in improving therapeutic efficacy in cancer immunotherapy. Highlights the contribution of targeting the TME in cancer immunotherapy.
Development of immunotherapy strategies targeting TME; Rilan Bai et al. [136].	Developing immunotherapy approaches that target the TME across different cancer types.	Precision immunotherapy remodels TME into a positive immune microenvironment. Challenges include a lack of research models and spatiotemporal heterogeneity.
Cancer: a mirrored room between tumor bulk and TME; Pablo Hernández-Camarer et al. [137].	TME and its role in the maintenance of a cancer stem-like phenotype via tumor metastasis, progression, invasiveness, and drug resistance. The paper discusses the importance of understanding the interaction between the TME and metastasis to improve clinical management of cancer.	Demonstrates the feasibility of reprogramming TAMs to an antitumorigenic state, showing potential for improving therapeutic outcomes.
Role of TME in cancer progression and therapeutic strategy; Qingjing Wang et al. [138].	Interaction between PD-1 and the TME, as well as ensuring cancer immunotherapy therapeutics. The paper mentions the inhibition of PD-1, PD-L1, and PD-L2, the construction of CAR-T, and tumor vaccines as popular therapeutic approaches.	TME plays a crucial role in cancer progression and therapeutic strategies. Cancer immunotherapy shows promising therapeutic effects in various cancers.

## 5. Conclusions

In conclusion, this review underscores the transformative impact of cutting-edge technologies in the field of oncology. By overcoming the limitations of traditional bulk sequencing, SCS and ST provide unprecedented insights into tumors. These techniques enabled the detailed characterization of cellular subpopulations and the mapping of crucial signaling pathways. Key pathways are VEGF and HIF-α, which are pivotal in promoting angiogenesis and ensuring the tumor’s blood supply, and CTLA-4 and PD-1/PD-L1 pathways, which are central to immune checkpoint regulation and enable tumors to evade immune surveillance by inhibiting T cell activity. Targeting these pathways has shown promise in enhancing antitumor immune responses. Despite this transformative potential, these techniques are not without drawbacks. The high cost, substantial data analysis requirements, and technical complexity pose significant challenges. In addition, sophisticated computational tools and models for interpreting the ST data are under development. Nevertheless, the integration of SCS and ST represents a paradigm shift in cancer research, offering the promise of more precise and personalized therapeutics.

## Figures and Tables

**Table 1:** Descriptions of some of the proteins involved in the TME, along with their functions.

S. No	Proteins in TME	Function	Pathways
01	Collagen	Influences prognosis, recurrence, and resistance in cancer [80].	p53 pathway, tumor necrosis factor (TNF) receptor 2/p38 MAPK signaling pathway
02	Fibronectin	Serves as a scaffold for matrix proteins and binding sites for the TME to function [81].	α5β1 pathway
03	Integrins	Mediate interactions between cell–cell and cell–ECM [82].	RAS/PI3K/AKT and RAF/MEK/ERK/mTOR pathway
04	Cadherins	Tissue homeostasis and cell–cell adhesions [83].	MAPK/ERK, MEK/ERK, Ras/MAPK, PI3K/AKT and ERK/MAPK pathway
05	TGF-β	It plays a role as a tumor suppressor and clears malignant cells by reducing growth and development [84].	JAK/STAT, PI3K/Akt, NF-κB, pathway
06	VEGF	It plays a key role in angiogenesis [85].	PI3-K/Akt, PI3K/mTOR, PI3K pathways
07	PD-1 and PD-L1	These frame the mechanism by which tumor cells achieve immune escape [86].	
08	CTLA-4	Inhibitor of T cell functions [87].	RAS pathway
09	Exosomes	Potentially affects the TME, remodels the ECM, and promotes vasculogenesis [88].	RAB, MAPK, P13-K/Akt, RAS, ESCRT-independent and ESCRT-dependent pathways
10	HIF-1α	Promotes the malignancy of the progression of tumors [89].	pVHL, FIH-1, Ras/Raf/MEK: Rat sarcoma/rapidly accelerated fibrosarcoma/MAPK/ERK kinase, Mdm2-p53-mediated ubiquitination and proteasomal degradation, Hsp90 pathways
11	Nucleolin (NCL)	NCL helps in chromatin remodeling, processes pre-RNA, drives rDNA transcription, and assembles ribosomes [90].	

**Table 2:** Studies using SCS and ST that reveal tumor heterogeneity, improve diagnostic precision, and guide targeted therapeutic strategies.

Study (Year)	Modality	Genes Involved	Diagnostic Contributions	Therapeutic Contributions
Navin et al., 2011 [95]	SCS	*ERBB2*, *MYC*, *TP53*	Preliminary single-cell DNA sequencing of breast cancer revealed subclonal copy number variation architecture, differentiating between punctuated and clonal evolution models.	Identification of CNV-driven oncogenes facilitates therapeutic targeting options for HER2+ breast cancer.
Patel et al., 2014 [96]	SCS (scRNA-seq)	*EGFR*, *PDGFRA*, *NF1*, *TP53*	Intratumoral heterogeneity was shown by single-cell transcriptomics, revealing different expression profiles. Distinguished between stem-like and differentiated states.	Resistance mechanism-related heterogeneity guides combination treatments aimed at several pathways.
Tirosh et al., 2016 [91]	SCS (scRNA-seq, melanoma)	*MITF*, *AXL*, *CD8A*, *PDCD1*	Detected immune evasion and two transcriptional states—MITF-high (proliferative) and AXL-high (invasive).	Revealed the reasoning for adaptive resistance to BRAF/MEK inhibitors; this data is used to guide patient classification and immunotherapy.
Zhang et al., 2016 [97]	SCS	*BCR-ABL1*, *JAK2*, *TET2*, *DNMT3A*	Monitored the clonal progression of leukemia, differentiating between driver and passenger mutations.	Enhanced precision therapeutics in CML/AML by facilitating the prognosis of therapeutic response and recurrence.
Chung et al., 2017 [92]	SCS + multi-omics	*PIK3CA*, *ESR1*, *TP53*	The incorporation of SCS with protein data was used to delineate genotype–phenotype heterogeneity.	The relationship between genotype and functional protein expression for therapeutic guidance.
Wang et al., 2023 [98]	ST (review)	Multiple datasets (methodological)	Condensed computational frameworks for the delineation of spatial heterogeneity in diagnostics.	Formulated a therapeutic stratification methodology that incorporates ST.
Chen et al., 2022 [99]	ST	*SOX2*, *OLIG2*, *GFAP*	Emphasized the geographic variability of stemness genes and their interactions with the surroundings.	Therapeutic insights into the targeting of stem cell-like populations inside the tumor microenvironment.
Lyubetskaya et al., 2022 [100]	ST	*PD-L1*, *CD274*, *CXCL13*, *IGHG1*	In situ characterization of B cell microenvironments and immunological checkpoint expression.	Contributed to the advancement of spatially informed immunotherapies and checkpoint inhibitors.
